# From Sensors to Sustainability: Integrating Welfare, Management, and Climate Resilience in Small Ruminant Farm Systems

**DOI:** 10.3390/ani15223240

**Published:** 2025-11-08

**Authors:** Maria Giovanna Ciliberti, Marzia Albenzio, Agostino Sevi

**Affiliations:** Department of Agriculture, Food, Natural Resources, and Engineering (DAFNE), University of Foggia, 71121 Foggia, Italy; maria.ciliberti@unifg.it (M.G.C.); marzia.albenzio@unifg.it (M.A.)

**Keywords:** small ruminants, climate change, precision livestock farming, animal welfare, milk production

## Abstract

**Simple Summary:**

Due to the increase in global demand for animal-derived products, it is essential to make livestock production more ethical and sustainable. This review focuses on the welfare of small ruminants in the context of climate change and heat stress. High temperatures negatively impact animals’ health, reproduction, and productivity. Precision livestock farming technologies, including sensors and automated systems, can detect in real time early signs of stress or disease and milk quality, allowing faster responses for predicting animal welfare. However, considering that animal responses to heat are complex, future models should include several environmental and physiological factors, such as wind, solar radiation, and body temperature measured with wearable or contact-free sensors. Integrating these data into automated systems could enable continuous monitoring and support timely interventions, such as nutritional adjustments or cooling strategies. Overall, combining climate adaptation, welfare management, and advanced digital tools offers a pathway toward more ethical, sustainable, and resilient livestock production systems.

**Abstract:**

In recent years, animal welfare has become a high priority in livestock production systems owing to the pressure to balance environmental sustainability, productivity, and ethics as demand continues to grow. This review presents the latest advances in small ruminant welfare, with emphasis on the effects of climate change, the main new innovative managerial and husbandry methods, and the use of precision livestock farming (PLF) technologies. In the first part, this review will examine how climate change is already re-shaping environmental and physiological conditions for farmed sheep and goats, with rising heat stress and negative impacts on both productive and reproductive performance. Secondly, more recent advances in small ruminant management will be presented, including improved housing systems, nutritional strategies, and behavioral monitoring, aimed at enhancing animal resilience and performance. Finally, particular focus will be given to the use of PLF tools for assessing milk quality and monitoring animal welfare. Evidence suggests that real-time monitoring technologies and sensor systems can accurately capture physiological and production parameters and provide an early sign of stress or health issues. Overall, the findings suggest that an integrated approach, combining climate adaptation strategies, welfare management, and the integration of precision technologies can serve as a key driver toward more ethical, sustainable, and resilient livestock production systems.

## 1. Introduction

### 1.1. Animal Welfare Concept

The concept of animal welfare can be traced back to the definition provided by Hughes [1], who emphasized the role of the environment in ensuring animals’ complete physical and mental well-being. According to this view, an animal is considered to be in a good state of welfare when it is in harmony with the surrounding environment. Later, Carpenter [2] introduced the influence of human intervention, suggesting that animals should be able to live in or adapt to human-made environments without experiencing pain. However, animal welfare is affected by more than just environmental factors. Exposure to physiological stressors, such as the postpartum period, calving, dry period, and growth and fattening phases, and psychological stressors, among which include human–animal interactions and separation of the offspring from the mother, can negatively impact welfare, leading to distress. Based on these definitions, animal welfare can be defined as the provision of living conditions that allow animals to express all their natural behaviors. The importance attributed to animal welfare varies across countries due to differences in religion, economic development, education, and cultural attitudes. Furthermore, animal welfare is not only a concern within farms but also a subject of growing interest among scientists and the general public. In many cases, there may be a conflict between improving animal welfare and maintaining the profitability and sustainability of animal production [3]. Therefore, when considering animal welfare, it is essential to address both the physical health and the psychological well-being of animals.

### 1.2. Biology of Stress in Livestock Farming and Precision Livestock Farming

**Figure 1:**
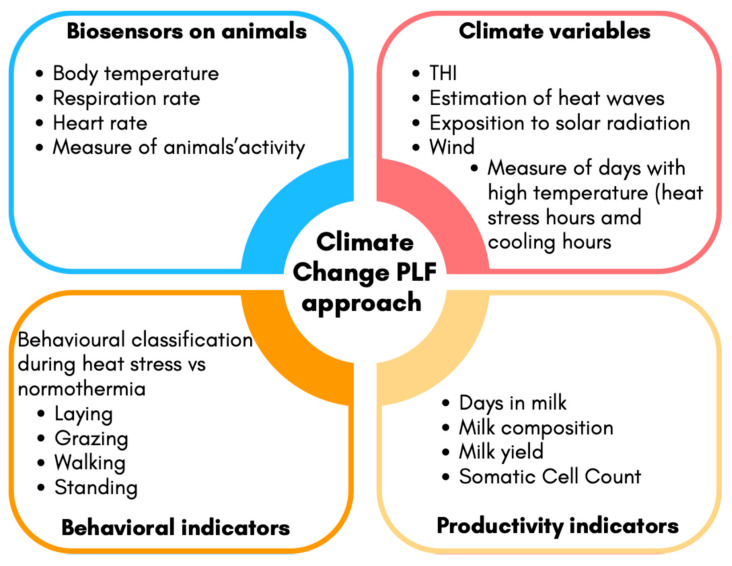
Proposed precision livestock farming (PLF) approach to assess and manage animal welfare under climate change scenario. The system integrates real-time data from the following: (1) biosensors mounted on animals, measuring physiological parameters such as activity level, respiration rate, heart rate, and body temperature; (2) climate variables, including data on ambient temperature and humidity, enabling the calculation of the temperature–humidity index (THI), detection of heat waves, and quantification of heat exposure hours and high-temperature days; (3) productive performance indicators, including milk yield and composition, days in milk, and somatic cell count, as recorded in farm management software; (4) behavioral indicators, enabling classification between heat stress-related and normothermia behaviors. These data streams feed into real-time algorithms or machine learning models, which estimate the animal’s welfare status and support decision-making by the farmer. The system aims to restore homeostasis by enabling timely interventions that ensure adequate energy allocation for maintaining biological functions and adaptive responses.

A stress response begins when the central nervous system perceives a potential threat to homeostasis. Factors such as trauma, noise, heat, humidity, environmental factors, and restriction of feed and water induce stress in animals. Regardless of the magnitude of the threat, the mere perception of stress is the critical event that activates a cascade of four coordinated biological responses: behavioral, autonomic nervous system, neuroendocrine, and immune responses. Moreover, the changes that occur are divided into three periods, i.e., alarm, resistance, and exhaustion [4,5]. From the alarm and resistance period to stressors or threats, the secretion of pituitary hormones by the hypothalamic–pituitary neuroendocrine system is altered; therefore, the broad and long-lasting effect of those hormones on immune competence, reproduction, metabolism, and behavior is modified, leading to a reduction in or an alteration of previous biological responses and a variety of syndromes. However, the intensity and display of these syndromes differ among individuals. Glucocorticoids such as cortisol alter glucose metabolism via glycogenolysis, increasing the blood concentration of glucose. In this context, blood pressure and mental activity increase, and fatty acids are mobilized from adipose tissue. Also, the autonomic sympathetic system stimulated by stressors releases epinephrine from the medulla of the adrenal gland, which exerts a cortisol-like effect, increasing body temperature, respiration rate, and depth. The physiological response can be of short or long durations. However, in the case of stressors exposure is prolonged, or when the effects are severe, body defense mechanisms become insufficient, and an exhaustion period occurs [3]. The biological cost during the exhaustion period becomes a significant burden to the body, during which animals enter the prepathological and pathological stage of stress [6]. The prepathological state can be defined as a condition in which biological functions are sufficiently altered to place animals at risk of developing pathologies in the broadest sense. This concept is essential, as it encompasses not only the onset of disease due to stress but also metabolic shifts that allow animals to grow, reproduce, or exhibit changes in normal behavior [6]. Heat stress in livestock can be defined as a multidimensional physiological challenge arising when the environmental thermal load exceeds the animal’s capacity for heat dissipation, triggering a cascade of adaptive responses. According to Moberg’s concept of the summation of a stressor, heat stress can interact synergistically with concurrent physiological demands, such as lactation and pregnancy, amplifying the metabolic burden. In this context, thermoregulation imposes a significant energy cost, resulting in altered metabolic prioritization that may compromise productive and reproductive functions, and this potentially drives the organism into a pre-pathological state since the distress period can last until the animal restores biological functions to pre-stress levels (in the absence of heat stress). Moreover, the recent heat wave trends in Europe [7], mainly driven by climate change, cause perturbations in the large-scale circulation system. These changes lead to the increased intensity and frequency of hot extreme events, such as heat waves, characterized by extremely warm and dry periods, resulting in a high number of fatalities and significant financial losses, especially in the agriculture and health sectors. Therefore, measuring or defining stress and distress in animals living in the current changing scenario is a challenge, and this can be addressed using the precision livestock farming (PLF) approach by combining data from sensors, automated systems, data analytics, and artificial intelligence systems integrated with farm management software. According to Berckmans [8], the primary aim of PLF is to “manage individual animals by continuous real-time monitoring of health, welfare, production/reproduction, and environmental impact.” The term continuous refers to data being measured and analyzed every second, 24 h a day, 7 days a week. These technologies are designed to automatically detect deviations from normal patterns and trigger warning alerts for farmers, allowing for timely interventions. This ultimately supports both animal welfare and farm efficiency. In the context of climate change, PLF systems should include sensors capable of detecting environmental fluctuations, such as those affecting the temperature–humidity index (THI), which combines temperature and humidity into a single index [9], along with monitoring behavioral patterns typically associated with thermal discomfort (e.g., changes in feeding, resting, or respiration behavior). In this regard, developing integrated prediction systems would be highly valuable. These systems should combine data from biosensors on animals (e.g., body temperature, respiration rate); environmental sensors (e.g., THI); and productivity indicators (e.g., milk yield, milk composition, somatic cell count) to better detect early signs of thermal discomfort and provide a more sensitive measure of the animal’s biological response to environmental stressors. However, as demonstrated in numerous studies, the response to stressors is highly individual-specific. Even within the same herd and under identical environmental conditions, some animals may be more affected than others—potentially entering a pre-pathological state before the onset of overt clinical symptoms. This concept is fundamental in the development of PLF technologies, as it requires viewing the animal as a complex, individually different, time-varying, and dynamic (CITD) system [8]. Each animal is biologically unique, responds to stimuli in a temporally variable manner, and continuously interacts with its environment through both innate and adaptive regulatory mechanisms [10]. When these mechanisms are overwhelmed by internal or external challenges, the animal enters a state of stress, triggering its physiological stress response systems. The ability to detect and interpret these signals in real time is one of the core strengths and promises of next-generation PLF systems. Modelling animal responses during heat stress, in particular, reflects many key features of complex systems. First, it is a classical complex dynamic process observed on a daily basis, where the interaction among variables results in continuous changes over time. Second, it includes feedback loops due to the animal’s endogenous ability to regulate heat dissipation and body temperature. Third, the response is nonlinear, as the accumulation of heat stress can lead to disproportionate physiological effects. Thus, this makes the cause–effect relationships among variables difficult to predict. Moreover, biological and physiological time delays are often present, resulting in lag effects in which performance declines may only become apparent after heat exposure has occurred. Taken together, these aspects confirm that the impact of heat stress on animal responses should be approached as a complex system problem [11]. Figure 1 illustrates a possible PLF framework for monitoring and managing animal welfare under climate change conditions, incorporating these dynamic and multifactorial characteristics along with possible indicators that are measurable through PLF technologies.

## 2. Climate as Welfare Modifier

How organisms respond to a changing environment is an emerging problem that is becoming a greater focus of basic research, especially in light of global change. Therefore, to address the question of how animals are able to cope with heat stress, the use of animal-based indicators in detecting heat stress response directly is of particular importance [12,13,14]. Indeed, improved animal welfare during exposure to heat stress depends, in part, on the timely assessment of heat loads to which the animals are exposed [15]. When environmental conditions become too extreme, heat stress may lead to drastic changes in biological functions [16]. Indeed, a decrease in feed intake efficiency and utilization and disturbances in water, protein, energy, and mineral balances; enzymatic reactions; hormonal secretions; and blood metabolites have been demonstrated. Moreover, a reduction in fecal and urinary water losses and an increase in respiration, panting, and sweating have been found, which are particularly limited in sheep due to wool cover [16]. These physiological imbalances are particularly important, as the cumulative effect of multiple stressors can divert metabolic resources away from essential biological functions, ultimately reducing the productivity and economic efficiency of livestock systems. Therefore, it is crucial to find reliable methods for measuring animal welfare in climate change conditions. In this regard, the animal husbandry sector has long been limited by the lack of labor- and time-saving measurement methods for animal-based indicators [17]. Based on this gap, in recent years, many thermal indices have been developed, starting from easily accessible weather data for reducing multi-dimensional thermal stressors, that could be connected to animal-based indicators.

### 2.1. Thermal Indexes

A good indicator of the degree of stress caused by weather conditions is conventionally accepted to be represented by the THI, which combines ambient temperature (T) and relative humidity (RH). Moreover, THI is considered the common thermal index due to its easy access and acceptable performance. Several formulas have been proposed in order to determine the THI in livestock, as reported in Table 1. These formulas have also been applied in sheep studies for the evaluation of the degree of heat stress exposure.

The Kelly and Bond [21] formula combines the maximum temperature (expressed in °C) and the average of relative humidity (%). In lactating ewes, a THI higher than 80 and a prolonged exposure to maximum air temperature over 30 °C cause heat stress exposure by decreasing milk production and quality [40,41] and their reproductive performance [42]. Moreover, moving from a THI of 60–65 to 72–75 induces a significant reduction in sheep performance by 20% [43]. Variations in body temperature, respiration rate, and heart rate, which tend to increase under thermal stress, can be used to measure an animal’s reaction to heat stress [44]. Therefore, the correlation between physiological parameters with THI could comprise useful information regarding heat tolerance and adaptability in indicating breeds for a particular region. In a study carried out by Kumar et al. [45], Munjal sheep reared in a subtropical climate demonstrated positive correlations among respiration rates (RRs), rectal temperatures (RTs), and THIs in both lambs and adult sheep, with an increase in the value of RR and RT during hotter periods of the day (afternoon: 2:00–4:00 pm). Their study concluded that it is crucial to design targeted management practices to support the physiological well-being of animals during periods with a high temperature and humidity index, ensuring optimal health and productivity. However, in the context of climate change and the tropicalization of climate, it could be crucial to modify the thermal index based on specific environmental conditions, like those used in arid and semiarid regions and also those used in the Mediterranean region [26]. This modification was introduced by Buffington et al. [26], and it modifies the THI by Thom [18], determining the BGHI index with higher R2 values with respect to RT and RR in dairy cows. Then, Barbosa and Silva [46] proposed a TCI for sheep, which resulted in higher R2 values correlating with RT and RR rather than BGHI and THI. The aforementioned indices are practical tools for determining an area’s general climate; they involve local meteorological measurements of air temperature and relative humidity, wind speed, mean radiant temperature, and solar radiation. However, the variables and their coefficients in a specific index must be in accordance with the physiological mechanisms of the heat exchange of the animals under consideration. Mascarenhas et al. [47] proposed a novel THI thermal index for native sheep in in tropical regions: “TSI = 24.153 − (0.0523 × AT) + (0.746 × BGT) + (4.104 × Vp)”, where air temperature (AT, °C), black globe temperature (BGT, °C), dew point temperature (DPT, °C), relative humidity (RH, %), and air partial vapor pressure (Vp, kPa) were included. The novel thermal index TSI registered high correlations with physiological variables and correlations between the already existing indexes.

### 2.2. Physiological Indices

#### 2.2.1. Respiration Rate

During exposure to excessive heat, the regulation of body temperature is supported by respiratory dynamics that facilitate heat dissipation [48]. In sheep, one of the primary physiological responses to heat load is an increase in RR [49]. In particular, changes in respiratory dynamics under heat stress are typically characterized by two distinct phases of panting [50]. The first phase of panting involves rapid, shallow breaths, which lead to an increase in RR and a moderate rise in respiratory volume. The second phase is marked by slower but deeper respirations, which are often accompanied by open-mouthed panting and a greater respiratory volume than that observed in the first phase [50]. For this reason, RR is considered one of the most important indicators of thermoregulatory response to heat in sheep [51]. The normal respiration rate range for sheep is between 16 and 30 breaths per minute, as related to the breed, age, and body condition score [52]. The common method for assessing RR is through the manual counting of flank movements for either 30 s or 1 min [20,53], which is a robust method; however, it requires the presence of a person, and it cannot be fitted in real time with environmental conditions, which are essential to monitor the physiological responses to heat stress. Recent advances in biometric traits, instead of traditional methods for identifying individual animals, have increased interest due to the development of convolutional neural networks (CNNs) [54,55,56]. A number of CNNs have been developed for animal recognition tasks [57] and specifically for biometric identification [40,41], and these have been significantly improved for accuracy with the development of VGG, ResNet, and EfficientNet architectures [57,58,59,60]. With this approach, a recent study [61] assessed the viability of a non-contact method for estimating respiratory rates in sheep models in laboratory conditions for continuous animal welfare assessment. The system was designed to support continuous animal welfare monitoring using, in the first stage, RGB (visible light) videos and, subsequently, near-infrared (NIR) imaging for detailed chest and body analyses. The recorded videos underwent a segmentation step, during which the thoracic regions used for RR estimation were extracted using the Detectron2 framework [62]. RR was then estimated by analyzing chest motion during respiratory cycles in resting animals, specifically tracking the expansion and contraction of the thorax. This was achieved by identifying and following specific markers in the x and y axes across video frames [63]. The entire process involved five main steps (preprocessing, motion tracking, filtering, component selection, and respiratory curve observation). Despite some limitations, the models exhibited reduced performance when only part of the animal was visible. The study demonstrated the feasibility of using video-based technologies for non-invasive, simultaneous RR monitoring. Furthermore, the approach could potentially be integrated with behavioral analysis algorithms capable of detecting postures (e.g., standing vs. lying) using video analysis, enabling a more comprehensive real-time assessment of animal welfare. Another automated alternative to manual RR measurement was validated by Fulghesu et al. [64]. This method started with the biometric identification of the animal for the 3D characterization of its surface and volume, using a geometric modeling approach based on solid equations and starting from a cylinder used for the limbs and truncated cones for neck, thorax, and abdomen identification. Then, RR was measured, with visual counts of respiratory acts detected in two 1 min videos. In this model, the air temperature (AT), season, and hours were considered as the main effects, while the body surface/volume ratio, estimated based on biometrics measures, was included as a covariate. As expected, the results demonstrated that the diurnal variability of the RR can be explained by AT (56%), while 1% of variability was explained by the season (winter = 0 and summer = 1 in the equation), and 1% was explained by the body surface/volume ratio. The authors concluded that the season’s effect can be quantified via +12.8 act/minute in summer vs. winter seasons. Another study [65] applied an analogous approach using computer vision for a non-contact evaluation of RR, analyzing the inhalation and exhalation of animals. The method proposed the implementation of automated computer vision algorithms for analyzing breathing videos, machine learning models for obtaining critical biometrics from recorded RGB, and infrared thermal videos of sheep for assessing heat stress. Interestingly, it was designed as a user-friendly AI system that has been combined with information incorporating blockchain technology for control purposes. All previously mentioned methods have limitations, as they are based on tracking algorithms and often require animals to be restrained in cages under non-natural conditions in order to obtain complete and continuous respiratory signals. Despite these conditions, the automated monitoring of RR through a computer vision approach is a rapidly growing and evolving field. It represents one of the most promising approaches for reducing physical contact with animals while enabling continuous, non-invasive observation in more realistic and welfare-friendly conditions.

#### 2.2.2. Body Temperature

A crucial indicator for evaluating thermal stress in sheep is core temperature, reflecting the function of the main internal organs such as the heart, brain, and viscera [66]. Rectal temperature is considered the most accurate estimation of core body temperature, typically ranging from 38.5 to 39.5 °C, but the use of a thermometer is invasive, time-consuming, and subject to variability due to operator technique. Moreover, it can induce severe stress in animals and increased metabolic heat production associated with the flight response. All these factors can affect the reliability and accuracy of RT recorded by a thermometer [67,68], especially under heat stress exposure. In addition, it is not applicable when the continuous assessment of the body temperature of free-range animals or those in an extensive grazing system is required [69]. Over the years, several alternative methods have been proposed to overcome these limitations. These include indwelling thermal sensors, such as rectal probes, which allow for remote temperature monitoring without removing animals from their production or grazing environments [70]. Similarly, vaginal temperature sensors have shown a high correlation with RT measurements [71,72]. Other technologies have also been developed to monitor body temperature, such as surgically implanted devices [73], endo-ruminal boluses equipped with temperature sensors [74], and infrared thermography. The use of ruminal boluses to evaluate heat stress requires certain adjustments, as ruminal temperatures may differ from core body temperature by approximately 2 °C [75]. Infrared thermography (IRT) is a non-invasive and promising technology for assessing stress, welfare, and heat stress exposure in livestock [76,77]. It has been applied to determine thermal thresholds [78] and predict the effects of heat stress on reproductive performance [79]. Accurate use of IRT requires careful calibration, especially in free-range systems, where changes in meteorological parameters can affect the transmission and reception of infrared radiation [66]. Additionally, the cleanliness and dryness of the animal’s coat are critical, as dirt or moisture can alter emissivity and compromise accuracy [80]. In sheep, fleece length significantly influences heat exchange at the skin level [81], affecting the interpretation of peripheral temperatures [82]. Therefore, the presence or absence of wool must be considered when applying IRT, as the skin is an active thermoregulatory organ. Moreover, different body regions yield varying degrees of correlation with RT and are influenced by ambient conditions [83], but due to physiological differences in vasoconstriction and vasodilation, multiple measurement sites should be used [84]. Thermal windows, such as the eye, udder, or abdomen, are directly connected to the autonomic nervous system and dissipate heat efficiently [85,86], and different areas (ears, eyes, udders, hooves, vulva, anus, and abdomen) have been measured by IRT [87]. Among those areas, eye temperatures are used in estimating sheep core temperatures, which are highly correlated with RT and behavioral changes [88,89]. Overall, IRT offers a fast and effective method for screening large numbers of animals with minimal restraint [90], supporting the shift from manual to automated monitoring systems [91]. Nevertheless, it requires standardized protocols, including camera distance, angle, ambient temperature, light, and wind control, and it remains relatively costly and labor-intensive [14,76]. To address the limitations of IRT in predicting RT, recent studies have explored its integration with machine learning techniques [91]. Advanced models based on artificial neural networks (ANNs) can detect complex, nonlinear patterns between infrared data and biological responses [91,92,93]. These models improve over time through training procedures that adjust parameters to enhance predictive accuracy [93]. Fuentes et al. [65] and Joy et al. [91] have successfully applied ANN-based systems using IRT to monitor heat stress exposure, combining machine learning techniques with IRT and THI.

## 3. Automatic Milking System and Its Impact on Animal Welfare

Milking management is a key factor in small ruminant farming systems, with a significant impact on animal welfare, health, and milk production. Elements such as the animals’ adaptation time to machine milking, pre-parturition training in the milking parlor, the number of lambs per ewe, and the type of milking (manual or mechanical) can critically influence animal behavior and stress levels. In particular, stress related to milking is due to both genetic factors and previous handling experience by humans; therefore, reducing emotional and physical stress can help improve both animal health and productivity. Specifically, two main mechanisms explain how stress affects lactation: the local mechanism [49] and the systemic mechanism [6]. The first consists of the activation of the HPA axis, increasing cortisol levels in the blood by stimulating the release of plasmin activators in the mammary cistern, thus triggering the degradation of β-casein and producing the 1-28 β-casein fragment. This fragment blocks ion channels in the mammary epithelial cells, inhibits lactose and ion secretion, and ultimately reduces milk yield. In the systemic mechanism, ACTH secretion from the pituitary gland is implicated, which stimulates the adrenal cortex to produce glucocorticoids (such as cortisol). In previous studies, cortisol has been shown to reduce milk synthesis by blocking glucose uptake in the mammary gland [94]. A collateral effect is the inhibition of prolactin production due to dopamine release from the hypothalamus, leading to reduced milk output and a temporary metabolic energy surplus. This is linked to a spike in glucocorticoids, followed by increased insulin and fat tissue activity. Prolonged stress can negatively affect milk yield, particularly in late lactation, due to elevated leptin levels, which inhibit the positive effect of IGF-1 on the mammary gland. It has been demonstrated that calm ewes produce more milk than nervous ones [95], and gentle handling helps animals adapt and improves milking efficiency. Rough handling increases residual milk and milking time by inhibiting oxytocin release through stress-induced catecholamines [96,97]. In primiparous ewes, stress hormone levels (adrenaline, noradrenaline, and cortisol) are higher shortly after lambing, but they decrease over time as animals adapt to machine milking [98]. Training ewes to enter the milking parlor before weaning led to lower somatic cell counts in early lactation, though cortisol levels were not significantly affected, suggesting that the training period may have been too short to reduce stress effectively [99]. Machine milking, when performed correctly, does not reduce milk yield or quality compared to hand milking and may even improve udder health and milk hygiene by reducing somatic cell and bacterial counts [100,101]. However, poor milking practices can compromise milk hygiene. The transition from suckling to machine milking is a sensitive period marked by immune suppression and increased mastitis risk [102]. Proper hygiene in housing and milking procedures is essential. During the peripartum period, ewes show significant changes in immune response and IL-6 and IgG levels, which serve as indicators of physiological and nutritional stress, particularly influenced by the number of lambs expected [103]. Sheep carrying multiple gestations experience a greater reduction in immune function during the peri-partum period compared to those with single pregnancies. Consequently, they are more vulnerable to pathogens and require stricter hygiene control during housing and milking.

### 3.1. Technological Aspect of Milking System in Small Ruminant

From a technological point of view, three main components of the milking system directly influence milk ejection and animal welfare: vacuum level, pulsation, and milking unit. Their balance is crucial for the proper functioning of the system. However, as working vacuum increases, the milk flow rate may also rise, potentially exacerbating mammary gland disorders. In small ruminants, inappropriate vacuum settings or unstable vacuum levels [104,105,106,107] are associated with higher somatic cell counts and greater susceptibility to mastitis. Nevertheless, it is generally recommended that the vacuum be kept as low as possible without compromising the complete emptying of the udder or extending milking time. For low-line systems in good operating conditions, a vacuum level between 36 and 38 kPa is advised [108]. A prototype milk line for dairy ewes developed under the BENOLAT project operated effectively at 28 kPa [109]. Besides the absolute value, vacuum stability is also essential for animal welfare, and its instability is often a consequence of poor system designs (e.g., inadequate vacuum reserve or incorrect milkline dimensions) or improper operations (e.g., faulty pulsation, flawed milking routines). Therefore, regular monitoring of vacuum fluctuations during mechanical milking is a practical procedure for assessing the technical and managerial quality of milking operations. Pulsation is another crucial aspect for maintaining animal welfare during machine milking, preventing teat congestion and edema, minimizing mammary infections, and alleviating pain and discomfort [110] by modulating vacuum pressure beneath the teat [111]. Improper designs or configurations can lead to significant vacuum fluctuations and increase susceptibility to mastitis [112]. With regard to the milking unit, the length of the milk tube and liners with sufficient elasticity are essential to guarantee animal welfare during the milking procedure. Therefore, continuous and automated monitoring of key milking parameters represents a promising approach to optimize system performance, ensure animal welfare, and reduce human error in the milking process of small ruminants.

### 3.2. Milking Parlor Crucial Aspects and New Technologies

The milking parlor plays a crucial role in managing several key aspects of flock health, such as monitoring sheep milk parameters and identifying subclinical mastitis [113]. The milking routine and cluster detachments in small ruminants were recently reviewed by Dzidic et al. [114]. The use of automatic cluster removers significantly reduces overmilking and the resulting stress on mammary glands, thus lowering somatic cell count (SCC) levels. However, a recent innovation in the milking parlor is the integration of sensors for early mastitis detection. Changes in milk electrical conductivity, caused by altered electrolyte concentrations due to cell damage, are highly correlated with SCC, an important indicator of udder health. The change in conductivity can be measured with both conductivity sensors [115] and optical sensors based on infrared light scatter [116]. Both spectrophotometry and light scatter technologies can also be applied to assess milk quality traits such as acidity and coagulation properties [117]. Evaluating coagulation characteristics from bulk tank milk is essential for estimating the economic efficiency of cheese production and for selective breeding based on milk quality. For instance, Jiménez [118] demonstrated that casein contents can serve as indicators for milk quality control in breeding programs. Fourier transform mid-infrared (FT-MIR) spectroscopy has been tested to predict coagulation and acidity traits in sheep bulk milk, but it showed limited accuracy for predicting milk coagulation properties and acidity. Nonetheless, FT-MIR spectroscopy remains a promising screening tool for distinguishing between early-, mid-, and late-coagulating milk. Further studies are encouraged to explore its application at the industry level to identify milk with coagulation properties suitable for cheesemaking, thereby improving processing efficiency [117]. From a technological standpoint, the introduction of automatic vacuum shut-off systems in dairy sheep milking has helped reduce physical stress during milking, minimize the risk of over-milking, and lower labor requirements. These systems operate based on time limits (2–3 min) and milk flow-rate thresholds (below 100–250 g/min) [113]. Modern milking parlors are also equipped with individual animal feeders that deliver precise amounts of feed to each sheep, offering a precision feeding solution. This technology is well-established and commercially available from several manufacturers, such as Milkline^®^ (https://www.milkline.com/en/53-in-parlour-feeding-system, accessed on 1 February 2023). More recently, augmented reality has been tested as a novel management tool in a milking parlor. While promising, this technology currently relies on proprietary animal tagging systems and is dependent on quick response (QR) coding for functionality [119].

## 4. Continuous Animal Behavior Assessment

Animal behavior assessment represents an essential approach to understanding welfare issues and health status, particularly during exposure to heat stress. In extensive farming systems, the continuous monitoring of grazing behavior allows for improved feeding strategies and greater production efficiency [120]. With this purpose, recent studies have proposed multiple technological solutions that can be grouped according to several key points. From a technological perspective, wearable and non-invasive devices such as accelerometers, acoustic sensors, and computer vision systems have been widely applied to evaluate feeding behavior. Triaxial accelerometers, particularly when positioned on the jaw, enable accurate differentiation between activities such as biting, chewing, and ruminating [121,122]. Acoustic sensors, in contrast, classify feeding behaviors by detecting sound patterns associated with ingestion and mastication, although accuracy can be influenced by respiration and individual variability [123,124,125]. Computer vision techniques extract features from video data, such as head position and body posture, to classify behaviors, offering a low-cost alternative, though one requiring controlled environments to minimize background interference [126,127].

A second integrative perspective concerns the combination of multiple sensors to improve behavioral classification and environmental interpretation. Wearable devices, such as accelerometers, gyroscopes, magnetometers, and GPS units, have been used to identify activities like walking, standing, lying, or aggressive behaviors [128,129,130,131]. Sensor placement (e.g., ears, neck, back) and sampling frequency significantly affect the performance of behavioral classification [132,133]. Evidence suggests that multi-sensor frameworks can enhance the accuracy of behavioral assessments. For instance, Di Virgilio et al. [134] integrated geographic information systems with accelerometers, magnetometers, temperature sensors, and GPS to determine feeding patterns, energy expenditure, and environmental interactions, while Horie et al. [135] applied a similar method to evaluate the influence of climatic conditions on sheep grazing behavior. However, despite the high precision achieved by combining accelerometers, GPS, and weather station data for applications such as lambing detection [136,137,138], these systems often result in increased energy consumption and computational costs [139,140,141]. Therefore, multi-sensor solutions may be best suited for preliminary testing to identify the most appropriate and cost-effective technology for specific behavioral targets.

The artificial intelligence (AI) model of data analysis highlights the growing role of machine learning and deep learning techniques in behavioral assessment. Kleanthous et al. [136] applied deep transfer learning using accelerometer data to recognize sheep activities, demonstrating that CNNs combined with transfer learning enable efficient data acquisition and management of sensor variability. The ability of transfer learning to adapt pre-trained models to new devices or datasets reduces both time and resource requirements, although algorithms developed for one sensor type are not yet directly transferable to others. Further developments include the use of support vector machines for posture classification [142] and Mask R-CNN for detecting standing and lying behaviors in dense flocks with high accuracy [143]. Deep learning has also been applied to address data imbalance in recognizing rare behaviors, such as aggression or biting, showing promising results [144]. Additional approaches, such as thermal imaging and depth cameras, have been introduced for behavior monitoring in enclosed environments, providing robust detection even under variable lighting conditions, though high costs still limit their widespread application [145].

Finally, analyzing management perspectives, integrating all these technologies could support real-time decision-making and welfare monitoring in both intensive and semi-intensive systems. The recently proposed ReproBehaviorNet model [146] exemplifies this trend by mapping age- and sex-specific behavioral data to biological functions and AI-based processes. This framework integrates wearable sensors, video tracking, and machine learning to identify key reproductive and maternal behaviors such as estrus expression, mating performance, and maternal care. Overall, non-invasive monitoring technologies represent a promising tool for improving sheep farm management. Future research should focus on enhancing reliability, adaptability to real conditions, and resilience to both individual and environmental variability while also establishing standardized analytical and data analysis approaches for behavioral data interpretation.

## 5. Conclusions

As emerged from our review, methods for obtaining heat-strain-related data can be broadly categorized into direct measurements and predictive modeling. While both approaches have been discussed in the previous section, it is essential to further explore the potential of predictive modeling for assessing the long-term effects of heat stress, such as the impact of heat stress on milk production and the duration of heat exposure [147]. Looking ahead, future research is likely to focus on algorithm designs, particularly in combination with artificial intelligence technologies. For instance, deployable ML models in animal breeding offer promising applications not only for genetic improvement but also for the early prediction of body weight, disease detection, and other health or performance indicators. Integrating such data with genomic information could enable the development of predictive models that are applicable across a wide range of environmental conditions and farm settings [148]. One example of an ML application in sheep is the prediction of maternal success for lamb survival. In a study focusing on maternal physiological, genetic, and environmental factors, data from both native and crossbred prolific ewes in a high-altitude, cold-climate region were analyzed. The random forest algorithm achieved the highest accuracy (67.2%), with a kappa statistic of 0.41 and the lowest mean absolute error (0.59), demonstrating its effectiveness in predicting mothering scores based on dam characteristics, birth conditions, and lamb attributes [146]. The decision tree model identified time of lambing, lamb genotype, and need for lambing assistance as key decision points for classifying maternal ability. A similar study utilized the boosting algorithm, achieving an accuracy rate of 92.5% in classifying lambs based on survival status until weaning. The key predictive features included birth weight, month of birth, number of ewes per shepherd, floor space per ewe, and birth rank group [149]. These findings suggest that both individual animal traits (e.g., birth weight and type) and farm management factors (e.g., stocking density, labor distribution) are critical for developing strategies to reduce lamb mortality.

To date, no studies have validated ML-based predictive models specifically targeting sheep performance during heat stress exposure to improve animal welfare and productivity. Given the complexity of biological responses to heat stress, future predictive models should incorporate multiple physiological and environmental variables. Existing thermal indices may need to be redefined to account for environmental stressors, such as wind and solar radiation. In addition, predictive models should integrate temporal indicators, including heat waves, the frequency of high-temperature days, and cumulative heat stress or cooling hours. Furthermore, the inclusion of physiological parameters, such as respiration rate, rectal temperature, surface temperature, and heat rate captured via wearable or non-contact sensors, should be integrated into automated milking systems. This integration would support a more comprehensive and continuous real-time management system for predicting herd health and productivity under heat stress conditions to support timely and long-term management strategies (i.e., precision nutritional plan or cooling intervention).

## Figures and Tables

**Table 1:** The temperature–humidity index formula commonly applied to quantify the degree of heat stress in sheep.

THI Formula	Degree of Heat Stress	References
THI = [0.4 × (Tdb °C + Twb °C)] × 1.8 + 32 + 15	Value 74 or less = normal; 75 to 78 = alert; 79 to 83 = danger status; ≥84 = emergency [18].	[18]
THI = 0.4 (Td + Tw) + 15	Value 71 to 74 = mild heat stress; 76.8 to 77.3 = moderate heat stress; 79 to 81 = severe heat stress [19].	[19]
THI = (1.8 × Tdb + 32) − (0.55 − 0.0055 × RH) × (1.8 × Tdb − 26)	Maximum air temperature over 30 °C and THI higher than 80 prevent lactating ewes from maintaining their thermal balance, thus inducing heat stress [20].	[21]
THI = (0.55 × Tdb °C + 0.2 × Tdp °C) × 1.8 + 32 + 17.5	Value < 68 = comfort; 68 to <72 = mild discomfort; 72 to <75 = discomfort; 75 to <79 = alert; 79 to < 84 = danger; > 84 = emergency [22].	[23]
THI = 0.72 (W °C + D °C) + 40.6	Value = 70 or less = comfortable; 75 to <78 = stressful; > 78 = extreme distress [24].	[24]
THI = [0.8 × ambient temperature (°C)] + [(% relative humidity/100) × (ambient temperature − 14.4)] + 46.4	Value of 72.0 ± 2.6: thermoneutral condition [25].	[26]
THI = db °F – [(0.55 − 0.55 × RH) (db °F – 58)]	Value < 72 = absence of heat stress; 72 to <74 = moderate heat stress; 74 to <78 = severe heat stress; 78 and more = very severe heat stress [22].	[27]
THI = db °C − {(0.31−0.0031 × RH) (db °C − 14.4)}	Value <22.2 = absence of heat stress; 22.2 to <23.3 = moderate heat stress; 23.3 to <25.6 = severe heat stress; 25.6 and more = extreme severe heat stress [22].	[28]
THI = dry bulb (°C) − 0.55 (1 − relative humidity) × (dry bulb − 14.4)	Value > or = 72 no heat stress; 73 to 77 = mild heat stress; 78 to 89 = moderate; <90 as severe [29].	[30]
THI = Tdb °C − [0.55 × (1 − RH)] × (Tdb °C − 14.4)	THI ≥23: decline in milk production [31].	[31]
THI = 9/5 × ((T × 17.778) − (0.55 − (0.55 × RH/100)) × (T − 14.444))	Value <72: thermoneutral conditions in winter; 76 to 78.5: from mild to moderate heat stress [32].	[32]
THI = td − (0.55 − 0.55RH) × (td − 58)	THI > 80 dramatically reduces milk production and alters milk composition in sheep [33].	[33]
THI = Td − {(0.31 − 0.31 × RH) (Td − 14.4)}	THI values above 25.6 are considered from severe to extreme heat stress, and THI below 22.2 is normally considered as comfortable [28].	[34]
THI = 0.81 db °C + RH (db °C − 14.4) + 46.4	Value ≤74 = normal; >74 to < 79 = alert; ≤79 to <84 = danger; ≥84 = emergency [35].	[35,36]
THI = Ta + 0.36Tdp + 41.5	THI values above 78 might cause severe discomfort in maintaining normal body temperature in animals [37].	[37]
THI = (1.8*AT + 32) − [(0.55 − 0.0055 × RH) × (1.8 × AT − 26)]	Value <68 = comfort; 68 to < 72 = mild discomfort; 72 to <75 = discomfort; 75 to <79 = alert; 79 to <84 = danger; >84 = emergency [22].	[38]

THI—Temperature–humidity index; Tdb—dry bulb temperature; Td—dry-bulb temperature; Tw—wet bulb temperature; Twb—wet bulb temperature; Tdp—dew point temperature; AT—air temperature; RH—relative air humidity; °C—degree centigrade; °F—degree Fahrenheit. Adapted by Sejian et al. [39].

## Data Availability

No new data were created or analyzed in this study. Data sharing is not applicable to this article.
